# Metabolomic analysis and antioxidant activity of wild type and mutant chia ( *Salvia hispanica* L.) stem and flower grown under different irrigation regimes

**DOI:** 10.1002/jsfa.11256

**Published:** 2021-05-04

**Authors:** Bruna de Falco, Laura Grauso, Alberto Fiore, Rocco Bochicchio, Mariana Amato, Virginia Lanzotti

**Affiliations:** ^1^ School of Science, Engineering & Technology, Division of Food & Drink University of Abertay Dundee UK; ^2^ School of Pharmacy, Centre for Analytical Bioscience University of Nottingham Nottingham UK; ^3^ Department of Agricultural Sciences University of Naples Federico II Naples Italy; ^4^ Scuola di Scienze Agrarie, Forestali Alimentari ed Ambientali Università della Basilicata Potenza Italy

**Keywords:** early flowering genotypes, total polyphenolic content, arbutin, danshensu, *myo*‐inositol, polyunsaturated fatty acids

## Abstract

**BACKGROUND:**

Chia (*Salvia hispanica* L.) is a functional food from Central America. Interest in it is growing rapidly due to the many health benefits from the seed. However, when chia is grown at high latitudes, seed yield may be low whereas a high stem biomass and immature inflorescences are produced. Little is known about the chemical composition and the properties of stems and flowers. In this work, the metabolite profile, the antioxidant activity, and the total polyphenol content of stems and inflorescences were evaluated in a factorial experiment with different chia populations (commercial black chia and long‐day flowering mutants G3, G8, and G17) and irrigation (100% and 50% of evapotranspiration).

**RESULTS:**

The results show the influence of irrigation and seed source on the antioxidant activity and total polyphenol content of chia flower and stem. Inflorescences exhibit higher antioxidant activity, suggesting their potential use as natural antioxidant. The mutants G3 and G8, at 50% irrigation, contained the highest amounts of compounds with nutraceutical value, especially within the flower. The mutant G17 showed lower antioxidant activity and polyphenol content compared to other seed sources but exhibited high omega 3 content in flowers but low in stems. This indicates that chia varieties should be chosen according to the objective of cultivation.

**CONCLUSION:**

These findings, indicating a close relation of metabolite content with irrigation and seed source, may provide the basis for the use of chia flower and stem for their nutraceutical value in the food, feed, and supplement industries. © 2021 The Authors. *Journal of The Science of Food and Agriculture* published by John Wiley & Sons Ltd on behalf of Society of Chemical Industry.

## INTRODUCTION

Chia (*Salvia hispanica* L.) is a herbaceous plant from the *Lamiaceae* family, which contains over 900 species globally. It is native of southern Mexico and Central America.^1^ The fruit, often referred to as ‘seed’ of the chia crop was a staple in the diet of the Aztec and Mayan civilizations.^2^ In ancient Mayan, *chia* means strength indicating the vast benefits and large energy capacity of this crop. In the late 1900s chia was rediscovered and was diffused from its original growing areas to new areas of the world, e.g. Europe and Australia. The popularity of chia comes from the seeds being labeled as a superfood for their high content of nutraceuticals, which are related to physiological properties.^3^ Recent studies showed the mucilage released from seeds to possess useful rheological properties for food production^4^ and as soil stabilizer.^5^

Chia seeds have a high proportion by percentage of *α*‐linolenic acid (60%), linoleic acid (20%), carotenoids and phenols such as caffeic acids. They contain fiber at 34–40% and protein with a percentage of 19–23%, which is considered high compared to many other edible seeds and high antioxidant activity has been reported.^6^, ^7^, ^8^ Other applications of the seed include the addition to animal feed, enriching the final product with polyunsaturated fatty acids (PUFA); for example, eggs, poultry, and rabbit. This has been observed in both cow feed and chicken feed.^9^, ^10^

*Salvia hispanica* was originally a short‐day flowering crop with a threshold of around 12 daylight hours but breeding efforts have produced long‐day flowering mutants that can viably produce seeds at a high latitude.^2^ Mutants G8 and G17^2^ have been highlighted as containing the highest levels of beneficial bioactive compounds compared to the other mutants.^11^ These therefore have a strong potential to be cultivated at higher latitudes and with great interest for the food and feed industry.

Nevertheless, if original chia sources are grown at high latitudes, they accumulate a high vegetative biomass and inflorescences in which seeds do not have time to mature, therefore seed yield is very low.^12^

Currently, chia is not used to its fullest potential as recent research shows that plant parts other than seeds contain nutraceuticals.^7^ High levels of PUFAs and essential oils have been found in shoots, which have therefore been proposed as feed^10^, ^13^ and antimicrobials.^14^ Many organs of plants in the *Lamiaceae* family have nutraceutical and pharmacological properties: sage (*S. officinalis)* leaves have antioxidant, anti‐inflammatory and antibacterial activity and they are used against diabetes, Alzheimer's, and cancer; clinical trials confirm antinociceptive, hypolipidemic, and memory‐enhancing effects.^15^, ^16^ Sage flowers constituents include alkaloids, carbohydrates, fatty acids, glycosidic derivatives, phenolic compounds, and terpenes; resin from flowers is used against dental caries and in cosmetics.^17^

Chia leaves have been shown to contain hydroxycinnamic acids, flavonoids, mainly represented by apigenin and luteolin glycosides, and the uncommon acetyl vitexin and acetyl orientin never found before in the *Lamiaceae* family.^7^ Leaf volatiles were found, such as essential oils constituted mainly of sesquiterpenes.^14^

With the broad aim of obtaining the chemical profile of plants of great interest to the food, feed, and supplement industries, we analyzed stems and flowers of different seed sources of chia. Plants selected for the study were a commercially available black chia from Mexico and three long‐day flowering mutants (named G3, G8, and G17). A metabolomic analysis based on gas chromatography and mass spectrometry (GC–MS) and the evaluation of antioxidant activity and polyphenol content were performed. We also aimed to evaluate the effect of irrigation regime on the production of nutraceuticals.

## MATERIALS AND METHODS

### Chemicals and reagents

The reagents used for the analysis, namely anhydrous methanol, 2,2′‐Azinobis‐(3ethylbenzothiazoline‐6‐sulfonic acid) diammonium salt (ABTS), methoxyamine hydrochloride and *N*‐methyl‐*N*‐trimethylsilyltrifuoroacetamide (MSTFA), cycloleucine, and heptadecanoic acid were obtained from Sigma‐Aldrich (Gillingham, UK). The anhydrous sodium carbonate, Folin–Ciocalteu's reagent and gallic acid were obtained from Fischer Scientific (Loughborough, UK).

### Plant materials

Plant materials were obtained from an experiment where eight treatments were tested with three replications. Treatments were established from the factorial combination of the following factors:

Factor 1: Seed sources of *Salvia hispanica* L. with four levels:

Level 1: Mexico, a commercial black chia Mexico, obtained from Eichenhain–Hofgeismar–DE;

Levels 2–4: G3, G8, and G17: three long‐ day flowering mutant genotypes, obtained as described in Jamboonsri *et al*.^2^ and used in this study through agreement between the University of Kentucky (US) and the University of Basilicata (Italy).

Factor 2: Irrigation with two levels:

Level 1: V100 = non limiting water supply, where water was supplied twice weekly to replace all lost water as determined by weighing the pots.

Level 2: V50 = irrigation with 50% of the water supplied to the V100 treatment.

For each of the three replications of treatments plants were sown in a 0.5 L plastic pot and grown with average T = 24 °C. The soil was a silty loam with the following characteristics: sand (50–2000 μm) 43.6%, silt (2–50 μm) 34.2%, clay (<2 μm) 22.1% with 12 h day length to obtain inflorescences from all seed sources.

Plants were collected at the 10% flowering stage and stems were cut at the soil level and separated from leaves and inflorescences (called ‘flowers’ below).

### Extraction procedure

Extraction procedure took place in July 2018. Samples were ground using a mortar and pestle to reach a fine texture. Samples (1.5 g) were then extracted with 15 mL of water/methanol mixture at a 1:1 ratio and 15 mL of dichloromethane, covered, and mixed with a magnetic stirrer for 30 min at room temperature. The mixture was centrifuged at 3000×*g* for 30 min; then, the aqueous and organic fractions were separated accurately. Extraction and subsequent centrifugation have been repeated with the interphase. Both fractions were dried under vacuum (Rotavapor R‐114, Büchi, Switzerland) at 30 °C to obtain dry extracts. The dried samples were stored at 4 °C until analysis. Validation of the extraction protocol has been obtained by using a standardized sample preparation protocol previously developed and applied for chia seeds analysis.^11^, ^18^ This allowed building compound libraries that enabled effective compound identification and efficient dereplication. This protocol was based on two analytical methods complementary for polarity allowing a broad range of organic compounds to be identified. One sample preparation was used for polar compounds and another sample preparation was used for non‐polar compounds.

### Sample preparation and GC–MS analysis

To obtain volatile and stable compounds, the polar extracts were derivatized before GC–MS analysis. In GC–MS‐based metabolomic methods, the derivatization procedure of the polar extract is a two‐step process, beginning with oximation to reduce tautomerism of aldehydes and ketones, followed by OH, SH, and NH groups silylation.^19^ 1 mg mL^−1^ stock solution of each sample was prepared and an aliquot of this (150 μL) was mixed with internal standard (cycloleucine 1 mg mL^−1^, 10 μL) and evaporated to dryness in a vacuum centrifuge (Eppendorf Concentrator 5301). The sample was then oxymated with 50 μL of methoxyamine hydrochloride (20 mg mL^−1^) in pyridine at 60 °C for 45 min. Samples were then silylated with 150 μL MSTFA at 60 °C for 45 min. Derivatized samples were injected (1 μL) in a pulsed splitless mode into an Agilent‐7820A GC system with 5977E MSD operating in EI mode at 70 eV. The system was equipped with a 30 m x 0.25 mm id fused‐silica capillary column with 0.25 μm DB 5MS stationary phase (Agilent Technologies, UK). The analysis of polar compounds was performed under the following temperature program: 2 min of isothermal heating at 70 °C, followed by a 5 °C min^−1^ oven temperature ramp to 260 °C, and the 10 °C min^−1^ to 300 °C and held for 5 min. The system was then temperature equilibrated for 1 min at 70 °C before injection of the next sample.

The non‐polar extract was subjected to acid methanolysis before GC–MS analysis, so the lipids contained in the extracts were detected as fatty acid methyl esters (FAMEs), using heptadecanoate (17:0) as an internal standard.^19^ Then 20 μL of heptadecanoate (0.5 mg mL^−1^) was added to an aliquot of dissolved sample in *n*‐hexane. Samples were then evaporated in a fume cupboard. Fatty acids were esterified with methanol in the presence of an acidic catalyst (1 mL of MeOH/HCl 93:7) in a sealed vessel at 50 °C overnight. After the reaction, the sample was neutralized with a solution of NaOH 1 N, evaporated under nitrogen flow; then, FAMEs were extracted with 1 mL of hexane.^20^ Non‐polar extracts were separated on a 30 m × 0.25 mm capillary column with 0.25 μm DB‐23 stationary phase (Agilent Technologies, UK). The temperature program was set as follows: 1 min of isothermal heating at 50 °C, followed by a 25 °C min^−1^ oven temperature ramp to 175 °C and then 4 °C min^−1^ to 230, held for 5 min.

All spectra were recorded in the mass range 50–800 m/z. Both chromatograms and mass spectra were evaluated using the MassHunter Qualitative Analysis B.07.00 (Agilent Technologies, CA, USA). Mass spectra of all detected compounds were compared with standard compounds and with spectra in National Institute of Standard and Technologies library, NIST MS Search 2.2 Data were processed with the Automated Mass Spectral Deconvolution and Identification Software (AMDIS) (Agilent Technologies, CA, USA) software to deconvolute co‐eluting peaks. Artifact peaks, such as peaks due to derivatizing agents, were not considered in the final analysis. Peak areas of multiple peaks belonging to the same compound were summed together. The relative content of each metabolite was calculated from the Total Ion Chromatogram (TIC) using the computerized integrator and with cycloleucine as an internal standard.^21^ All determinations were performed in triplicate.

### Antioxidant activity

The free radical‐scavenging activity was determined as described by Re *et al*.,^22^ using the reduction of radical cation 2,2′‐Azinobis‐(3‐ethylbenzothiazoline‐6‐sulfonic acid) diammonium salt (ABTS). A mixture of 2.5 mL of 7 mmol L^–1^ ABTS and 44 μL of 140 mmol L^–1^ potassium persulfate was prepared and left in darkness overnight.

The stock solution of ABTS was diluted to 1:80 until the optical density (OD) reached a value between 0.7 and 0.8 nm when read at a wavelength of 734 nm. Samples were diluted to the appropriate concentration; polar stem samples with full irrigation (V100) were diluted to 4 mg mL^−1^ and with restricted irrigation (V50) to 1.5 mg mL^−1^ and polar flower samples both (V100 and V50) were diluted to 0.4 mg mL^−1^; ABTS solution (1 mL) was added to 100 μL of diluted samples and after 2.5 min the reduction was measured as the percentage of inhibition. Results were expressed in mmol Trolox equivalent antioxidant capacity (TEAC g^−1^) and a calibration curve ranging from 25 to 250 μmol L^–1^ of Trolox was used. All determinations were performed in triplicate.

### Total polyphenol content

Total polyphenolic content (TPC) was determined spectrophotometrically according to the method described by Singleton and Rossi^23^ with some modifications described below: 125 μL of diluted sample was mixed with 500 μL of distilled water and 125 μL of Folin–Ciocalteu reagent. After 6 min, 1.25 mL of a 7.5% sodium carbonate solution was added to the mixture and brought to a final volume of 3 mL with distilled water. Samples were then placed in complete darkness for 90 min at room temperature. The absorbance was read at 760 nm (Thermo Scientific Genesys 10S UV–visible spectrophotometer) and TPC was expressed in terms of gallic acid equivalents (GAE g^−1^). A calibration curve ranging from 0 to 100 μg mL^−1^ was used to quantify the TPC content in flower and stem extracts. All determinations were performed in triplicate.

### Statistical analysis

Relative quantification was carried out by integration of all the peak areas of the chromatogram profiles for each compound and normalizing all data to the internal standard of each sample. The ANOVA test was performed, and means were separated using the post‐hoc test of Tukey. Significance levels *P* < 0.05 were expressed as *P < 0.05. Significant difference was shown with the use of different lower‐case letters on diagrams. All statistical procedures were computed using the SPSS statistical package.

## RESULTS AND DISCUSSION

### Metabolite profile

**Figure 1:**
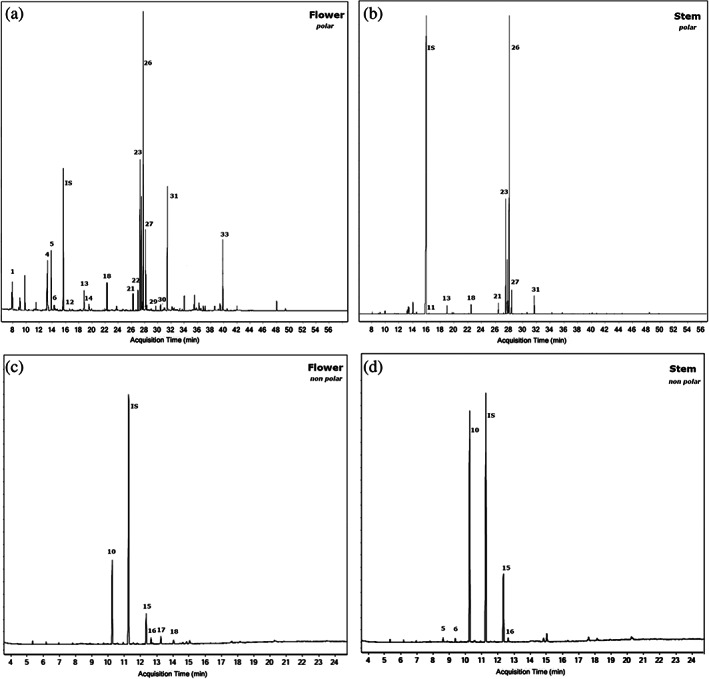
Total ion chromatograms (TIC) of chia flower (a) and stem (b) polar extracts where peaks correspond to numbering of compounds in Table 1. The TICs of chia flower (c) and stem (d) non‐polar extracts where peaks correspond to numbering of compounds in Table 2. Chromatograms of Mexico (50% irrigation) are taken as representative examples.

The whole metabolomic profile of *S. hispanica* L. was evaluated using GC–MS analysis. Metabolomic analysis of the silylated polar extracts resulted in the identification of 29 compounds in the flower and 23 compounds in the stem of the plant. Metabolomic analysis of non‐polar extracts identified 16 compounds in the flower and 15 compounds in the stem. All compounds from the polar and non‐polar extracts, with their respective retention times and m/z values, are listed in Tables 1 and 2, respectively. Some representative total ion chromatograms (TIC), in which major peaks are labeled with the respective compound numbers in Tables 1 and 2, are shown in Fig. 1.

**Table 1:** Polar metabolites identified in *Salvia hispanica* L. flower and stem by GC–MS

Peak number	RT	Compound^a^	Abbreviation	Molecular formula	*m/z*	Source^b^
1	7.84	Lactic acid, 2TMS	LA	C_9_H_22_O_3_Si_2_	73 117 147	F, S
2	9.02	l‐Alanine, 2TMS	Ala	C_9_H_23_NO_2_Si_2_	73 116 147	S
3	11.87	l‐Valine, 2TMS	Val	C_11_H_27_NO_2_Si_2_	73 144	S
4	13.27	Glycerol, 3TMS	GLY	C_12_H_32_O_3_Si_3_	73 147 205	F, S
5	13.84	l‐Proline, 2TMS	Pro	C_11_H_25_NO_2_Si_2_	73 142	F, S
6	14.39	Succinic acid, 2TMS	SU	C_10_H_22_O_4_Si_2_	73 147	F
7	14.75	Glyceric acid, 3TMS	GlyA	C_12_H_30_O_4_Si_3_	73 147,189,292	F
8	15.39	Fumaric acid, 2TMS	FU	C_10_H_20_O_4_Si_2_	73,75 147	F, S
9	15.54	l‐Serine, 3TMS	Ser	C_12_H_31_NO_3_Si_3_	73 156 204 218	F, S
10	15.70	Cycloleucine, 2TMS (int. standard)	cLeu (IS)	C_12_H_27_NO_2_Si_2_	73 156	F, S
11	16.35	l‐threonine, 3TMS	Thr	C_14_H_35_NO_2_SSi_3_	73 117 218	S
12	16.62	Hydroquinone, 2TMS	HQ	C_12_H_22_O_2_Si_2_	239 254	F
13	18.89	Malic acid, 3TMS	MA	C_13_H_30_O_5_Si_3_	73 147,233	F, S
14	19.55	l‐5‐Oxoproline, 2TMS	PCA	C_11_H_23_NO_3_Si_2_	73 156	F, S
15	19.74	*γ*‐Aminobutanoic acid, 3TMS	GABA	C_13_H_33_NO_2_Si_3_	73 147,174	F, S
16	20.38	4‐Propylbenzoate, TMS	4Pb	C_13_H_20_O_2_Si	147 177 221	F
17	20.50	2,3,4‐Trihydroxybutyric acid, 4TMS	tHB	C_16_H_40_O_5_Si_4_	73 147 205,292	F, S
18	22.30	Tartaric acid, 4TMS	TA	C_16_H_38_O_6_Si_4_	73 147,292	F, S
19	22.94	6‐Hydroxy‐2‐aminohexanoic acid, 3TMS	HAH	C_15_H_37_NO_3_Si_3_	73 128 246	F, S
20	25.89	l‐Sorbofuranose, 5TMS	SFu	C_21_H_52_O_6_Si_5_	73 147,217	F
21	26.25	Citric acid, 4TMS	CI	C_18_H_40_O_7_Si_4_	73 147,273	F, S
22	27.00	Quinic acid, 5TMS	QA	C_22_H_52_O_6_Si_5_	73 147,255,345	F, S
23	27.30	d‐Fructose‐MEOX (*anti*), 5TMS	Fru	C_22_H_55_NO_6_Si_5_	73 103 147 217	F, S
24	27.50	d‐Fructose‐MEOX(*syn*), 5TMS	Fru	C_22_H_55_NO_6_Si_5_	73 103 147 217	F, S
25	27.65	d‐Mannose‐MEOX, 5TMS	Man	C_22_H_55_NO_6_Si_5_	73 147 205,319	F
26	27.84	d‐Glucose‐MEOX, 5TMS	Glc	C_22_H_55_NO_6_Si_5_	73 147 205,319	F, S
27	27.60	d‐Galactose‐MEOX, 5TMS	Gal	C_22_H_55_NO_6_Si_5_	73 147 205,319	F, S
28	29.68	Ribonic acid, 5TMS	RA	C_20_H_50_O_6_Si_5_	73 147,292	F
29	29.94	Galactaric acid, 6TMS	GA	C_24_H_58_O_8_Si_6_	73 147,292,333	F
30	31.01	Danshensu, 4TMS	Dan	C_21_H_42_O_5_Si_4_	73 267	F
31	31.45	*myo*‐Inositol, 6TMS	Ins	C_24_H_60_O_6_Si_6_	73 147,217,305	F, S
32	32.45	Caffeic acid, 3TMS	CA	C_18_H_32_O_4_Si_3_	73 219 396	F
33	39.84	Arbutin, 5TMS	Arb	C_27_H_56_O_7_Si_5_	73 254 361	F
34	40.75	Sucrose‐MEOX, 8TMS	Sucr	C_36_H_86_O_11_Si_8_	73 217 361	S
35	43.35	Maltose‐MEOX, 8TMS	Mal	C_37_H_89_NO_11_Si_8_	73 204 361	S

^a^ TMS, trimethylsilyl derivatives; MEOX, methyloxime derivatives.

^b^ F, Flower; S, Stem.

**Table 2:** Non‐polar metabolites identified in *S. hispanica* L. flower and stem by GC‐MS

Peak Number	RT (min)	Compounds^a^	Abbreviation	Molecular formula	*m/z*	Source^b^
1	6.50	2,4,6,8‐Tetramethyl‐1‐undecene	tm Ude	C_15_H_30_	29,43,55	F, S
2	6.64	Oxalic acid, allyl pentadecyl ester	OxA (15:0)	C_20_H_36_O_4_	41,57,71	S
3	7.49	Oxalic acid, allyl hexadecyl ester	OxA (16:0)	C_21_H_38_O_4_	41,57,71	F
4	8.28	Phytol	Phy	C_20_H_40_O	43,81,95 123	F
5	8.61	Myristic acid ME	C14:0	C_15_H_30_O_2_	43,74,87	F, S
6	9.37	Pentadecanoic acid	C15:0	C_16_H_32_O_2_	74,87	S
7	9.38	12‐Methyl myristic acid ME	12 me C14:0	C_17_H_32_O_2_	43,74,87	F
8	9.71	2,4‐Di‐*tert*‐butylphenol	2,4‐DTBP	C_14_H_22_O	41,57 191 206	F, S
9	9.95	Nonanedioic acid	NA	C_13_H_22_O_4_	43,55,74	S
10	10.25	Palmitic acid ME	C16:0	C_17_H_34_O_2_	43,74,87	F, S
11	10.48	7‐*Z*‐Palmitoleic acid ME	C16:1n‐7	C_17_H_32_O_2_	41,55,74	F, S
12	10.53	9‐*Z*‐Palmitoleic acid ME	C16:1n‐9	C_17_H_32_O_2_	41,55,74	F, S
13	10.93	14‐Methyl palmitic acid ME	14 me C16:0	C_18_H_36_O_2_	43,74,87 284	F
14	11.27	Heptadecanoic acid ME (int. standard)	C17:0 (IS)	C_18_H_36_O_2_	43,74,87 143	F,S
15	12.34	Stearic acid ME	C18:0	C_19_H_38_O_2_	43,74,87	F, S
16	12.65	Oleic acid ME	C18:1	C_19_H_36_O_2_	41,55,74	F, S
17	13.26	Linoleic acid ME	C18:2	C_19_H_34_O_2_	55,67,81, 95	F, S
18	14.05	Linolenic acid ME	C18:3	C_19_H_32_O_2_	55,67,79,95	F, S
19	14.60	Hydrocinnamic acid,3,5‐di‐tert‐butyl‐4‐hydroxy‐ ME	HCtb	C_18_H_28_O_3_	277 292	S
20	14.82	Eicosanoic acid ME	C20:0	C_21_H_42_O_2_	43,74,87	F, S
21	23.54	Phosphatidylethanolamine	PE 16:0/16:0	C_37_H_74_NO_8_P	43,57,85,98	F

^a^ ME, methyl ester derivatives.

^b^ F, Flower; S, Stem.

### Polar phase

**Figure 2:**
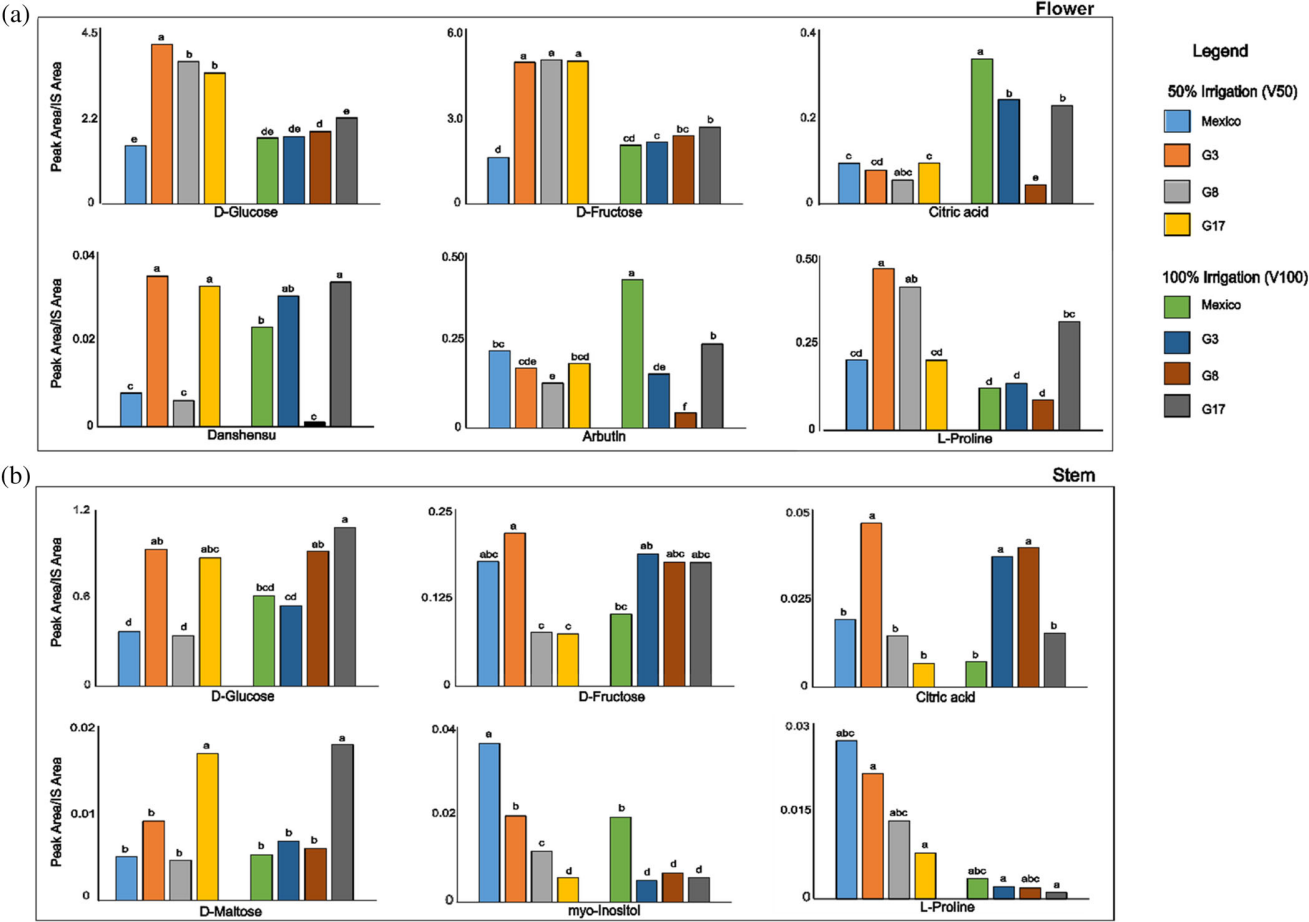
Effect of mutation and irrigation on some polar compounds identified in chia flower (a) and stem (b) polar extracts. Polar compounds were detected as trimethylsilyl and methyloxime derivatives by GC–MS. Different letters on the bars show significant differences between samples (*P* < 0.05) with the post‐hoc Tukey's test.

Carbohydrates were found to be the main class of compounds in all the aqueous extracts, glucose (Glc) and fructose (Fru) representing the major components. Most detected peaks contained a base peak at *m/z* 73, typical of silylated compounds, due to the [(CH_3_)_3_Si^+^] group. Analyses, performed in triplicate, showed significant differences for some metabolites among genotypes, but in most cases possible significant differences were masked by random differences in concentration.^2^ Metabolites whose amounts were significantly different among genotypes can be found in Fig. 2 and Tables S1 and S2 in the supporting information, where samples are noted with different letters when they showed a significant difference (*P* < 0.05) for the relevant metabolite.

In the flower, the most abundant compounds were sugars, including fructose, glucose, mannose (Man), and galactose (Gal). Abundant metabolites were also *myo*‐inositol (Ins), glycerol (GLY), and the glycoside arbutin (Arb). Other compounds identified were polyphenols and organic acids, including catechollactate (Dan), commonly named danshensu, citric acid (CI), lactic acid (LA), malic acid (MA), and the cyclic polyol quinic acid (QA) (Table S1 in the supporting information).

Metabolomic analysis of chia flowers showed the absence of disaccharides, which were instead present in stems and seeds. The amount of each monosaccharide was higher in mutants and lower in irrigated plants. The content of galactose was particularly high in mutant G17 (Table S1 in the supporting information). A positive effect of mutation on monosaccharides has been also reported in different genotypes of chia seeds.^24^

In general, the organic acids showed significant differences in concentration between genotypes; moreover, they were present in higher amounts in fully irrigated plants, except for mutant G8 (Table S1 supporting information).

The flower contained some interesting compounds commonly found in medical plants, such as arbutin, which is one of the most prescribed skin‐lightening and de‐pigmenting treatments in the world. It is found in many plant species and it is extracted from them for its topical skin uses. Arbutin is the *β*‐d‐glucopyranoside of hydroquinone. Both arbutin and hydroquinone are found in the flower extract; they act as inhibitors of tyrosinase activity and melanosome maturation and can therefore lighten skin or reduce unwanted skin pigmentation.^25^, ^26^ Danshensu is an active component in many *Salvia* plants, including *Salvia miltiorrhiza*, and has been used as herbal remedy in traditional Chinese medicine. Danshensu has been reported to have many medical benefits in the treatment of cardiovascular disease as it has been shown to promote blood circulation, improving blood flow in coronary arteries. Studies have provided evidence that danshensu presents potential anti‐tumor and anti‐angiogenesis properties.^27^, ^28^ Its content in the analyzed sample was significantly high in G3 and G17, while irrigation affected its concentration only in the commercial Mexico sample (Fig. 2).

A number of identified essential and non‐essential amino acids are listed in Table S1 in the supporting information; the most abundant amino acid is l‐proline (Pro). The GC–MS analysis showed a significant variation in the content of l‐proline related to genotype and irrigation only for mutants G3 and G8 (Fig. 2).

In the stem, the main identified sugars were glucose, fructose, and mannose. Glucose was the most abundant metabolite in all the analyzed polar samples and particularly in genotypes G3 and G17. The disaccharide maltose (Mal) had a high content in the mutant G17, with irrigation not affecting its amount in chia stems (Fig. 2). Besides carbohydrates, main compounds were *myo*‐inositol, the amino acid l‐proline, and the organic acid, citric acid, as reported in Table S2, in the supporting information. Polyols (*myo*‐inositol and glycerol) were detected in different amounts in all genotypes and lower in irrigated samples, except for G17. Some of the detected compounds have been known to possess beneficial effects and are commonly used by the supplement industry. For example, *myo*‐inositol is an insulin sensitizer, and it has shown good results in the treatments of insulin resistant polycystic ovarian syndrome with positive effects on serum progesterone levels.^29^ Essential and non‐essential amino acids have also been found, such as l‐valine, l‐threonine, l‐proline, and l‐alanine. Their amounts in chia stems were influenced by genotype and irrigation, being lower in mutants, and reaching the minimum amount in samples from plants subjected to 100% irrigation (Fig. 2, and Table S2 in the supporting information). These metabolites give many benefits to the human body; for example, l‐proline is fundamental in the development of collagen, a key molecule for healthy and operational joints and tendons.^30^ l‐Valine is also used in the supplement and fitness industry as component of the branched‐chain amino acids (BCAAs) because it is able to stimulate muscle protein synthesis and prevent muscle degradation after exercise.

*Salvia hispanica* stems also contained organic acids such as citric, tartaric, lactic, and malic acids. The genotype G3 was characterized by a significantly higher content of organic acids, except for lactic acid, which instead is particularly abundant in mutant G8. Organic acids were less abundant in samples from irrigated plants, although this trend was not always statistically significant (Table S2 in the supporting information).

### Organic phase

**Figure 3:**
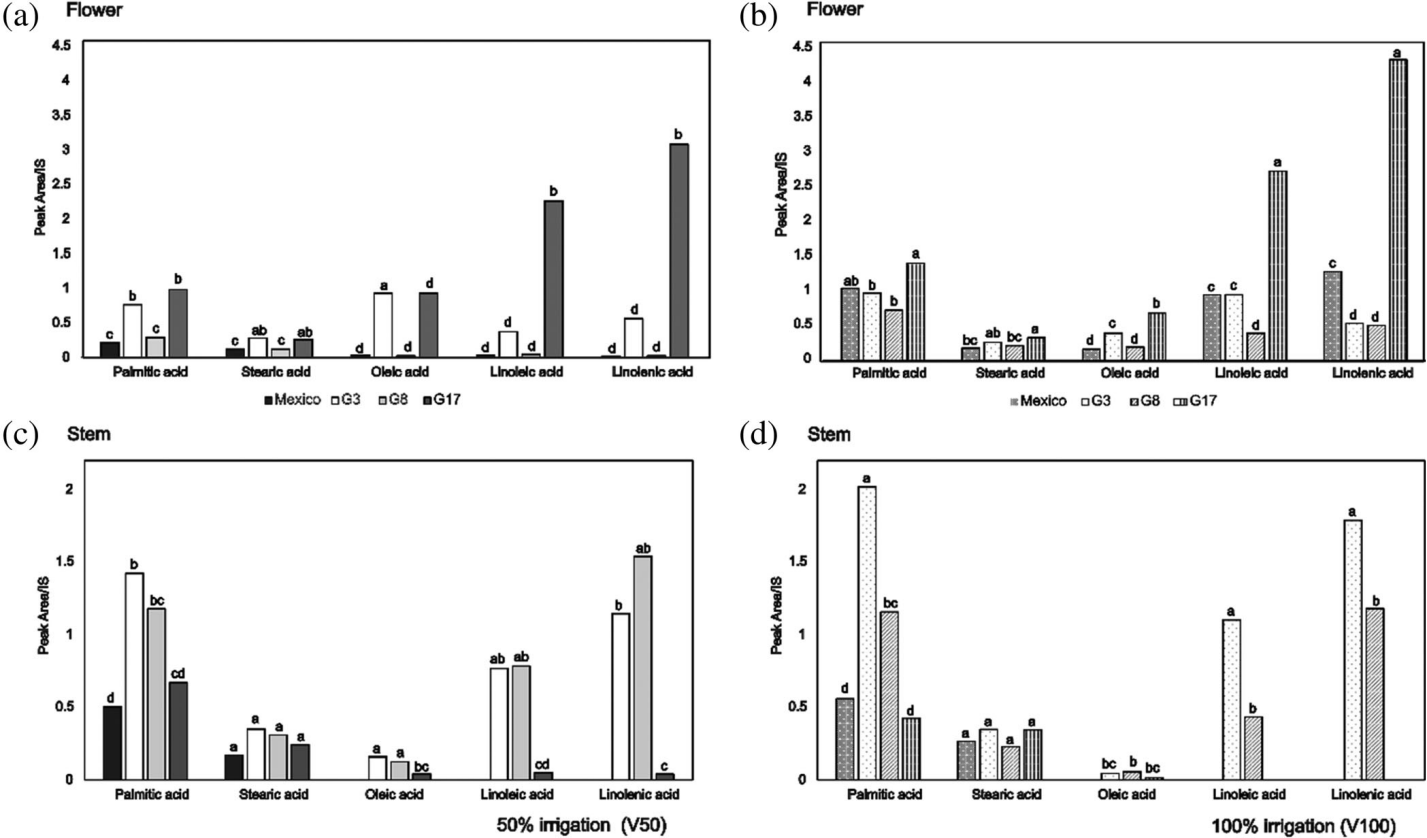
Effect of irrigation on chia fatty acids composition: (a) flower at 50% irrigation, (b) flower at 100% irrigation, (c) stem at 50% irrigation, and (d) stem at 100% irrigation. Fatty acids were detected in non‐polar extracts as methyl esters by GC–MS. Different letters on the bars show significant differences between samples (*P* < 0.05) with the post‐hoc Tukey's test.

The main metabolites in the organic extracts of flower and stem were saturated and unsaturated fatty acids. They were detected and are listed in Table 2 as FAMEs, obtained after a methanolysis reaction. Figure 3, and Tables S3 and S4 in the supporting information, show the relative quantification of fatty acids for each genotype at the two levels of irrigation. Palmitic, stearic, oleic, linoleic, and linolenic acids were found as the major fatty acids in both parts of the plant. Very interestingly, the composition of the fatty acids in the flower of G17 (both irrigation regimes) showed the same linolenic > linoleic > palmitic acid pattern as that reported for chia seeds.^11^ Conversely, G8 (both irrigation regimes), G3 (100% irrigation) and Mexico (50% irrigation) showed palmitic acid as the most abundant acid. As for organic extracts of the stem, palmitic acid was most abundant in all genotypes, except for G8 (50% irrigation) where linolenic acid predominates. Surprisingly, linoleic and linolenic acids were not detected in Mexico (both irrigation regimes) and G17 (100% irrigation) genotype, and they were found in very low concentration in G17 (50% irrigation) compared to other chia genotypes, although it is reported in the literature that the irrigation treatment affects the fatty acid composition of plants, data from stem extracts show a positive effect with significant differences only for linolenic and palmitic acid in G3. Data from flower extracts revealed a positive effect of irrigation for palmitic, linoleic, and linolenic acids in G8, G17, and Mexico (Fig. 3, Table S3 in the supporting information). Although some fatty acids increased with water supply, the oleic / linoleic acid ratio decreased in all the flower and stem genotypes. These data agree with the literature and follow Ivanov's rule: the amount of linoleic acid rises when the temperature decreases, in contrast with oleic acid.^11^, ^31^, ^32^ This trend can be ascribed to a thermal effect of water supply, keeping fully irrigated plants at a lower temperature.

Minor compounds have been identified in both stem and flower extracts. Some of them have been recognized by the Food and Drug Administration (FDA) as flavoring agents or adjuvants, such as myristic acid and phytol, an acyclic diterpene alcohol and a constituent of chlorophyll. Data from the literature report antioxidant activity for 2,4‐di‐*tert*‐butylphenol, detected in both extracts, and reaching the highest value in G8 and G3 genotypes.^33^, ^34^, ^35^

### Antioxidant activity

**Figure 4:**
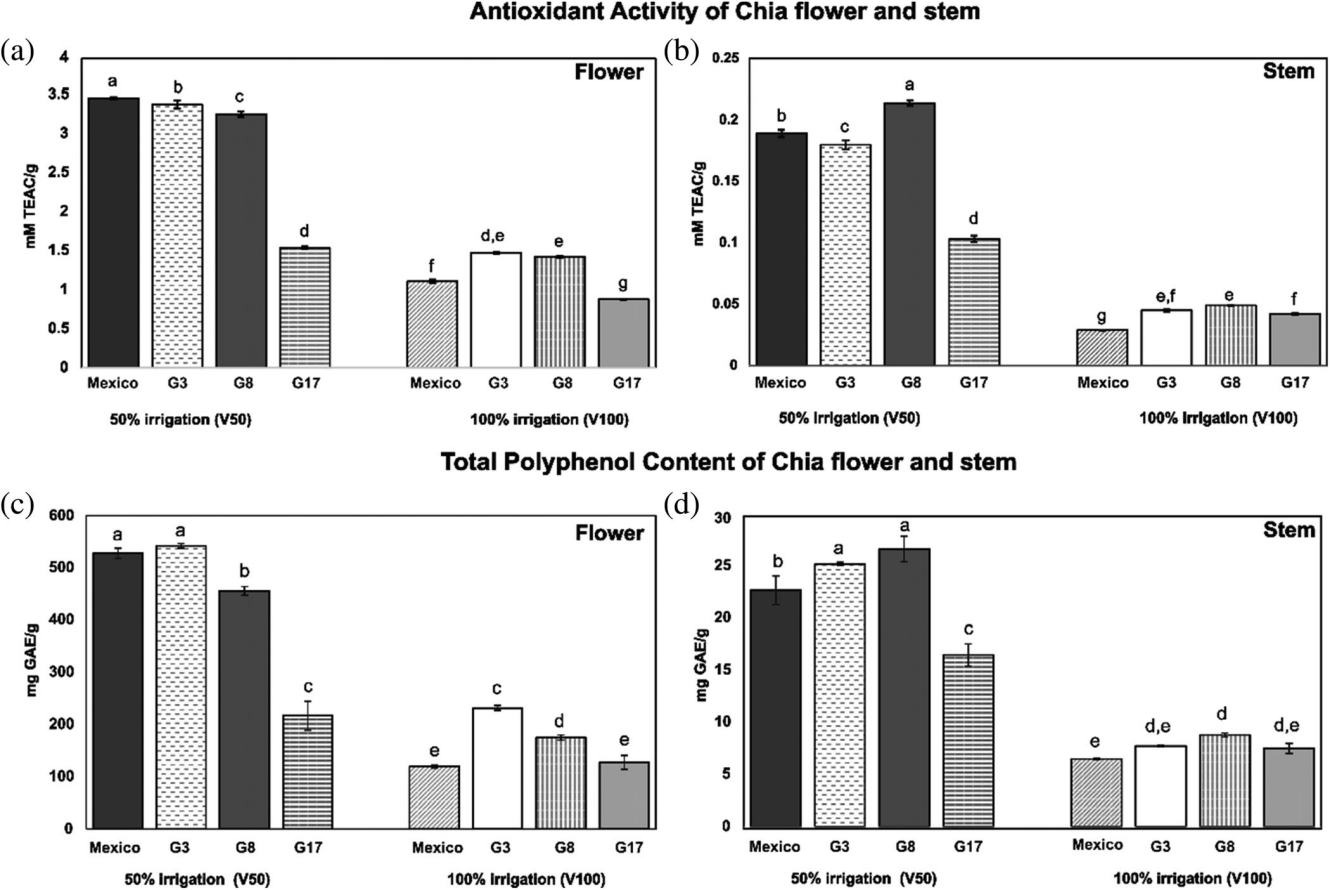
Antioxidant activity and TPC of chia flower (a and c, respectively) and stem (b and d, respectively) at different irrigation regimes (50% irrigation and 100% irrigation) shown as Trolox equivalent. Different letters on the bars indicate significant differences (*P* < 0.05) with the post‐hoc Tukey's test.

Results of antioxidant activity for both flowers and stems are shown in Fig. 4. Both stems and flowers always exhibit significantly lower values at 100% than at 50% irrigation. Values for flowers were highest, with a maximum of 3.4 ± 0.01 mmol L^–1^ TEAC g^−1^ in 50% Mexico, whereas the highest value for stems was found in G8 (50% irrigation) and amounted to 0.21 ± 0.00 mmol L^–1^ TEAC g^−1^. The G17 mutant showed significantly lowest values in many cases (Fig. 4).

### Total polyphenol content

The TPC results are shown in Fig. 4. Data indicate that the inflorescence contains a high proportion of total polyphenols compared to the stems. The highest amount was found in G3 and Mexico flowers followed by G8 (all of them at 50% irrigation). In general, it was observed that, in both plant compartments, total polyphenol content was reduced by full irrigation.

Zhang & Kirkham^36^ have shown that the response of antioxidants to water supply varies with plant type. A previous study by de Falco *et al*.^11^ on the chia seeds indicated significant differences in antioxidant capacity due to different irrigation levels. The data on chia flowers and stems confirm that the whole plant is affected by drought conditions and a larger antioxidant profile can be achieved by osmotic stress.

The scoring order of seed sources was different in stems than in flowers and in the different irrigation regimes. The G17 mutant exhibited lower levels of polyphenols than other seed sources at 50% irrigation, while in 100% irrigation it showed significantly lower values than G8 and G3 in flowers and was not significantly different from other seed sources in stems. The Mexico commercial source scored lowest in full irrigation, but it was the highest or second in range between seed sources at 50% irrigation. This interaction between irrigation and seed source is an important finding as it suggests that the choice of variety for best performance should be made according to growing conditions.

Our findings for flowers and stems are also partly consistent with findings on chia seeds in an experiment comparing two commercial seed sources and three mutants in a full irrigation regime,^24^ where commercial sources showed lower values of total polyphenol content and antioxidant activity than long‐day flowering mutants, as found in fully irrigated stems and flowers of our experiment. However, in the seed trial, G17 showed intermediate values between G3 and commercial sources, and it was identified as one of the most interesting mutants to consider for seed production, whereas in our experiment on stems and flowers, G17 was the lowest scoring by far in full irrigation.

This suggests a different allocation of antioxidant compounds in different plant parts for different seed sources.

### Relationship between antioxidant activity and Total polyphenol content

Pearson's correlation coefficient revealed a significant positive relationship between the antioxidant activity and total polyphenol content of flowers (r (24)=0.987, *P* < 0.001). A significant positive relationship was also found between the antioxidant activity and total polyphenol content of the stem (r (24)=0.987, *P* < 0.001]. The data on the high antioxidant activity of inflorescences parallels findings from GC–MS analysis showing a metabolic profile rich in phenols, whereas stems were rather rich in carbohydrates.

Many antioxidant compounds such as tocopherols, phytosterols, carotenoids, and phenols have been found in chia seeds^7^, ^37^ and leaves.^7^ Phenols include compounds such as chlorogenic and caffeic acids, myricetin, quercetin, and kaempferol.^38^ High antioxidant activity has therefore been reported for whole chia seeds or extracted oil,^7^ and mucilage.^4^

In our data the main phenolic compounds identified in the flower extracts of *S. hispanica* (Table 1, and Table S1 in the supporting information), are caffeic acid and danshensu, the former being an orally bioavailable, polyphenol, with many potential health applications. All phenols are key elements of a plant because they are free radical scavengers due to the presence of hydroxyl groups. Consequently, the content of plant total polyphenols directly contributes to its antioxidant activity. Our data agree with the literature, which report polyphenols as major antioxidant compounds in medicinal plants.^39^

Other compounds with antioxidant activity were found in chia flowers (Table 1, and Table S1 in the supporting information). In particular, it is to underline the high levels found for arbutin reported in the literature as a long‐lasting radical‐scavenger.^40^

Poly‐unsaturated fatty acids are also natural antioxidants normally found in chia seeds^7^ and in our data also in stems and flowers (Tables S3 and S4 in the supporting information).

The data clearly indicate that flowers are rich in antioxidants. This was already described for flowers of other *Salvia* species^41^ and it is probably due to the presence of additional phenols acting as pigments in the flowers. In our case, the high antioxidant activity of inflorescences was due to both phenolic and non‐phenolic compounds.

All 50% irrigation samples of the flower extract show antioxidant activity levels in the same range or even higher than that of the seeds,^11^, ^37^ suggesting that the flowers are the best source of natural antioxidants in the plant.

## CONCLUSIONS

As far as we are aware, this is the first report of the characterization of the metabolome of flowers and stems of *S. hispanica*. The comparison of the composition of four different seed sources at two irrigation treatments has shown that irrigation has a significant impact on metabolite content, antioxidant activity and polyphenol content with higher values found at 50% water supply compared to full irrigation. The results show that chia inflorescences exhibit high antioxidant activity levels and suggest they could be a more potent natural antioxidant than the plant seeds.

We identified the mutant G3 and G8 (50% irrigation) as containing the highest amounts of compounds with nutraceutical value, especially within the flower; it therefore has the highest potential for attracting interest from the food, feed, and supplement industries. The mutant G17 always scored low in antioxidant activity and polyphenol content compared to other seed sources but, on the other hand, it showed high omega‐3 content in flowers but low in stems.

Thus, this work shows that chia can be grown in areas outside its original ones without loss of bioactive metabolites and that chia varieties should be chosen according to the objective of cultivation. An interaction between irrigation and seed source was found. For instance, the commercial seed source compared with long‐day flowering mutants scored low in antioxidant content and activity with full irrigation but high with 50% water supply. This is an important finding as it has been shown elsewhere that the choice of chia variety for seed yield and quality should be made according to photoperiod sensitivity and agronomy. Our results add to that and indicate that, in choosing varieties for the content and activity of antioxidants in stems and flowers, irrigation regimes must not be neglected.

## Supporting information


**Table S1.** Quantification of detected compounds in the polar extracts of flowers.
**Table S2.** Quantification of detected compounds in the polar extracts of stems.
**Table S3.** Quantification of detected compounds in the non‐polar extract of flowers.
**Table S4.** Quantification of detected compounds in the non‐polar extract of stems.

## References

[1] Ixtaina V , Nolasco S and Tomás M , Physical properties of Chia (*Salvia hispanica* L.) seeds. Ind Crops Prod 28:286–293 (2008).

[2] Jamboonsri W , Phillips TD , Geneve RL , Cahill JP and Hildebrand DF , Extending the range of an ancient crop, *Salvia hispanica* L.—a new ω3 source. Genet Resour Crop Evol 59:171–178 (2012).

[3] de Falco B , Amato M and Lanzotti V , Chia seeds products: an overview. Phytochem Rev 16:745–760 (2017).

[4] Menga V , Amato M , Phillips TD , Angelino D , Morreale F and Fares C , Gluten‐free pasta incorporating chia (*Salvia hispanica* L.) as thickening agent: an approach to naturally improve the nutritional profile and the in vitro carbohydrate digestibility. Food Chem 221:1954–1961 (2017). 27979185 10.1016/j.foodchem.2016.11.151

[5] di Marsico A , Scrano L , Labella R , Lanzotti V , Rossi R , Cox L *et al*., Mucilage from fruits/seeds of chia (*Salvia hispanica* L.) improves soil aggregate stability. Plant Soil 425:57–69 (2018).

[6] Ayerza R , Oil content and fatty acid composition of chia (*Salvia hispanica* L.) from five northwestern locations in Argentina. J Am Oil Chem Soc 72:1079–1081 (1995).

[7] Amato M , Caruso MC , Guzzo F , Galgano F , Commisso M , Bochicchio R *et al*., Nutritional quality of seeds and leaf metabolites of Chia (*Salvia hispanica* L.) from southern Italy. Eur Food Res Technol 241:615–625 (2015).

[8] Romankiewicz D , Hassoon WH , Cacak‐Pietrzak G , Sobczyk M , Wirkowska‐Wojdyle M , Ceglińska A *et al*., The effect of chia seeds (*Salvia hispanica* L.) addition on quality and nutritional value of wheat bread. J Food Qual 2017:1–7 (2017).

[9] Ayerza R and Coates W , Influence of chia on total fat, cholesterol, and fatty acid profile of Holstein cow's milk. Rev Cient de UCES 10:39–48 (2006).

[10] Jamshidi AM , Amato M , Ahmadi A , Bochicchio R and Rossi R , Chia (*Salvia hispanica* L.) as a novel forage and feed source: a review. Ital J Agron 14:1297–1314 (2019).

[11] de Falco B , Fiore A , Bochicchio R , Amato M and Lanzotti V , Metabolomic analysis by UAE‐GC MS and antioxidant activity of *Salvia hispanica* (L.) seeds grown under different irrigation regimes. Ind Crops Prod 112:584–592 (2018).

[12] Bochicchio R , Rossi R , Labella R , Bitella G , Perniola M and Amato M , Effect of sowing density and nitrogen top‐dress fertilisation on growth and yield of Chia (*Salvia hispanica* L.) in a Mediterranean environment: first results. Ital J Agron 10:163–166 (2015).

[13] Peiretti PGF , Fatty acid and nutritive quality of Chia (*Salvia hispanica* L.) seeds and plant during growth. Anim Feed Sci Technol 148:267–275 (2009).

[14] Elshafie HS , Aliberti L , Amato M , de Feo V and Camele I , Chemical composition and antimicrobial activity of chia (*Salvia hispanica* L.) essential oil. Eur Food Res Technol 244:1675–1682 (2018).

[15] Ghorbani A and Esmaeilizadeh M , Pharmacological properties of *Salvia officinalis* and its components. J Tradit Complement Med 7:433–440 (2017). 29034191 10.1016/j.jtcme.2016.12.014 PMC5634728

[16] Hamidpour M , Hamidpour R , Hamidpour S and Shahlari M , Chemistry, pharmacology, and medicinal property of sage (salvia) to prevent and cure illnesses such as obesity, diabetes, depression, dementia, lupus, autism, heart disease, and cancer. J Tradit Complement Med 4:82–88 (2014). 24860730 10.4103/2225-4110.130373 PMC4003706

[17] Narayanan N and Lakshmi T , *Salvia officinalis* in dentistry. Dent Hypotheses 6:27–30 (2015).

[18] de Falco B , Incerti G , Pepe R , Amato M and Lanzotti V , Metabolomic fingerprinting of Romaneschi globe artichokes by NMR spectroscopy and multivariate data analysis. Phytochem Anal 27:304–314 (2016). 27437863 10.1002/pca.2632

[19] de Falco B and Lanzotti V , NMR spectroscopy and mass spectrometry in metabolomics analysis of *Salvia* . Phytochem Rev 17:951–972 (2018).

[20] Grauso L , Zotti M , Sun W , de Falco B , Lanzotti V and Bonanomi G , Spectroscopic and multivariate data‐based method to assess the metabolomic fingerprint of Mediterranean plants. Phytochem Anal 30:572–581 (2019). 31286588 10.1002/pca.2862

[21] Jonsson P , Gullberg J , Nordström A , Kusano M , Kowalczyk M , Sjöström M *et al*., A strategy for identifying differences in large series of metabolomic samples analyzed by GC/MS. Anal Chem 76:1738–1745 (2004). 15018577 10.1021/ac0352427

[22] Re R , Pellegrini N , Proteggente A , Pannala A , Yang M and Rice‐Evans C , Antioxidant activity applying an improved ABTS radical cation decolorization assay. Free Radic Biol Med 26:1231–1237 (1999). 10381194 10.1016/s0891-5849(98)00315-3

[23] Singleton VL and Rossi JA , Colorimetry of total phenolics with phosphomolybdicphosphotungstic acid reagents. Am J Enol Vitic 16:144–158 (1965).

[24] de Falco B , Fiore A , Rossi R , Amato M and Lanzotti V , Metabolomics driven analysis by UAEGC‐MS and antioxidant activity of Chia (*Salvia hispanica* L.) commercial and mutant seeds. Food Chem 254:137–143 (2018). 29548434 10.1016/j.foodchem.2018.01.189

[25] Sarkar R , Arora P and Garg K , Cosmeceuticals for hyperpigmentation: what is available? J Cutan Aesthet Surg 6:4–11 (2013). 23723597 10.4103/0974-2077.110089 PMC3663177

[26] Bang SH , Han SJ and Kim DH , Hydrolysis of arbutin to hydroquinone by human skin bacteria and its effect on antioxidant activity. J Cosmet Dermatol 7:189–193 (2008). 18789053 10.1111/j.1473-2165.2008.00387.x

[27] Zhang L , Chen L , Lu Y , Wu J , Xu B , Sun Z *et al*., Danshensu has anti‐tumor activity in B16F10 melanoma by inhibiting angiogenesis and tumor cell invasion. Eur J Pharmacol 643:195–201 (2010). 20621088 10.1016/j.ejphar.2010.06.045

[28] Cao H , Ding R , Li M , Yang M , Yang L , Wu J *et al*., Danshensu, a major water‐soluble component of *Salvia miltiorrhiza*, enhances the radioresponse for Lewis lung carcinoma xenografts in mice. Oncol Lett 13:605–612 (2016). 28356936 10.3892/ol.2016.5508 PMC5351344

[29] Nas K and Tuu L , A comparative study between myo‐inositol and metformin in the treatment of insulin resistant women. Eur Rev Med Pharmacol Sci 21:77–82 (2017). 28724173

[30] ChEBI, L‐proline (CHEBI:17203) , Ebi.ac.uk, (2019). Available: https://www.ebi.ac.uk/chebi/searchId.do?chebiId=CHEBI:17203 [1 May 2019].

[31] Aparicio R , Ferreiro L and Alonso V , Effect of climate on the chemical composition of virgin olive oil. Anal Chim Acta 292:235–241 (1994).

[32] Dag A , Ben‐Gal A , Yermiyahu U , Basheer L , Nir Y and Kerem Z , The effect of irrigation level and harvest mechanization on virgin olive oil quality in a traditional rain‐fed ‘Souri’ olive orchard converted to irrigation. J Sci Food Agr 88:1524–1528 (2008).

[33] Varsha KK , Devendra L , Shilpa G , Priya S , Pandey A and Nampoothiri KM , 2, 4‐Di‐tert‐butyl phenol as the antifungal, antioxidant bioactive purified from a newly isolated *Lactococcus* sp. Int J Food Microbiol 211:44–50 (2015). 26164257 10.1016/j.ijfoodmicro.2015.06.025

[34] Yoon MA , Jeong TS , Park DS , Xu MZ , Oh HW , Song KB *et al*., Antioxidant effects of quinoline alkaloids and 2, 4‐di‐tert‐butylphenol isolated from *Scolopendra subspinipes* . Biol Pharm Bull 29:735–739 (2006). 16595909 10.1248/bpb.29.735

[35] Choi SJ , Kim JK , Kim HK , Harris K , Kim CJ , Park GG *et al*., 2, 4‐Di‐tert‐butylphenol from sweet potato protects against oxidative stress in PC12 cells and in mice. J Med Food 16:977–983 (2013). 24074359 10.1089/jmf.2012.2739 PMC3833388

[36] Zhang J and Kirkham M , Antioxidant responses to drought in sunflower and sorghum seedlings. New Phytol 132:361–373 (1996). 26763632 10.1111/j.1469-8137.1996.tb01856.x

[37] Reyes‐Caudillo E , Tecante A and Valdivia‐López MA , Dietary fibre content and antioxidant activity of phenolic compounds present in Mexican chia (*Salvia hispanica* L.) seeds. Food Chem 107:656–663 (2008).

[38] de Falco B , Incerti G , Bochicchio R , Phillips T , Amato M and Lanzotti V , Metabolomic analysis of *Salvia hispanica* seeds using NMR spectroscopy and multivariate data analysis. Ind Crops Prod 99:86–96 (2017).

[39] Ravipati A , Zhang L , Koyyalamudi S , Jeong S , Reddy N , Bartlett J *et al*., Antioxidant and anti‐inflammatory activities of selected Chinese medicinal plants and their relation with antioxidant content. BMC Complement Altern Med 12:173 (2012). 23038995 10.1186/1472-6882-12-173 PMC3534023

[40] Takebayashi J , Ishii R , Chen J , Matsumoto T , Ishimi Y and Tai A , Reassessment of antioxidant activity of arbutin: multifaceted evaluation using five antioxidant assay systems. Free Radic Res 44:473–478 (2010). 20166881 10.3109/10715761003610760

[41] Yumrutas OA , Sokmen A and Ozturk N , Determination of in vitro antioxidant activities and phenolic compounds of different extracts of *Salvia verticillata* ssp *verticillata* and spp *amasiaca* from Turkey's flora. J Appl Pharm Sci 1:43–46 (2011).

