# Supplementary material for: Metabolomic analysis and antioxidant activity of wild type and mutant chia ( Salvia hispanica L.) stem and flower grown under different irrigation regimes
Source: J Sci Food Agric. 2021 May 4;101(14):6010–9. doi: 10.1002/jsfa.11256 (PMC8518854; doi:10.1002/jsfa.11256)
Supplement: Supplementary file 1 — Table S1. Quantification of detected compounds in the polar extracts of flowers. Table S2. Quantification of detected compounds in the polar extracts of stems. Table S3. Quantification of detected compounds in the non‐polar extract of flowers. Table S4. Quantification of detected compounds in the non‐polar extract of stems. [file JSFA-101-6010-s001.docx]

Metabolomic analysis and antioxidant activity of wild type and mutant Chia (*Salvia hispanica* L.) stem and flower grown under different irrigation regimes

Bruna de Falco^a, b,1^, Laura Grauso^c,1^, Alberto Fiore^a^, Rocco Bochicchio^d^, Mariana Amato^d^, Virginia Lanzotti^c,*^

^a^ *School of Science, Engineering & Technology, Division of Food & Drink, University of Abertay, Bell Street, DD1 1HG Dundee, Scotland, UK*

^b^ *Faculty of Pharmacy, University of Nottingham, University Park, Nottingham NG7 2RD, United Kingdom*

^c^ *Department of Agricultural Sciences, University of Naples Federico II, via Università 100, I-80055 Portici, Naples, Italy*

^d^ *Scuola di Scienze Agrarie, Forestali, Alimentari ed Ambientali, Università della Basilicata, viale dell'Ateneo Lucano 10, I-85100 Potenza, Italy*

Supplementary Data

**Table S1**. Quantification of detected compounds in the polar extracts of flowers.

**Table S2**. Quantification of detected compounds in the polar extracts of stems.

**Table S3**. Quantification of detected compounds in the non-polar extract of flowers.

**Table S4**. Quantification of detected compounds in the non-polar extract of stems.

**Table S1.** Quantification of detected compounds (peak area/internal standard area) in the polar extracts of flowers. Different letters show significant difference between samples (p<0.05).

|  | **MEXV50** |  | **G3V50** |  | **G8V50** |  | **G17V50** |  | **MEXV100** |  | **G3V100** |  | **G8V100** |  | **G17V100** |  |
| --- | --- | --- | --- | --- | --- | --- | --- | --- | --- | --- | --- | --- | --- | --- | --- | --- |
| **LA** | 0.092±0.007 | b | 0.099±0.002 | b | 0.061±0.002 | cd | 0.069±0.013 | cd | 0.116±0.003 | a | 0.057±0.002 | d | 0.071±0.005 | cd | 0.075±0.002 | c |
| **GLY** | 0.331±0.010 | a | 0.449±0.030 | a | 0.378±0.037 | a | 0.344±0.073 | a | 0.350±0.019 | a | 0.417±0.041 | a | 0.335±0.244 | a | 0.361±0.024 | a |
| **Pro** | 0.207±0.002 | cd | 0.475±0.015 | a | 0.421±0.004 | ab | 0.206±0.151 | cd | 0.125±0.009 | d | 0.139±0.003 | d | 0.089±0.003 | d | 0.321±0.010 | bc |
| **SU** | 0.004±0.000 | g | 0.022±0.002 | e | 0.014±0.002 | f | 0.024±0.001 | e | 0.088±0.003 | a | 0.059±0.002 | c | 0.049±0.002 | d | 0.079±0.004 | b |
| **GlyA** | 0.007±0.000 | c | 0.016±0.000 | b | 0.015±0.002 | b | 0.014±0.000 | b | 0.023±0.001 | a | 0.023±0.002 | a | 0.015±0.000 | b | 0.022±0.001 | a |
| **FU** | 0.001±0.000 | d | 0.002±0.001 | cd | 0.003±0.000 | bc | 0.002±0.000 | d | 0.002±0.000 | cd | 0.005±0.000 | b | 0.007±0.000 | a | 0.002±0.000 | cd |
| **Ser** | 0.027±0.001 | c | 0.027±0.001 | c | nd |  | 0.029±0.002 | bc | 0.031±0.001 | b | 0.037±0.002 | a | 0.012±0.001 | e | 0.016±0.000 | d |
| **HQ** | 0.012±0.000 | b | 0.015±0.001 | a | 0.007±0.000 | c | 0.003±0.000 | d | 0.006±0.002 | c | 0.007±0.000 | c | 0.001±0.000 | d | 0.002±0.002 | d |
| **MA** | 0.133±0.005 | d | 0.212±0.009 | c | 0.190±0.003 | c | 0.182±0.012 | cd | 0.370±0.015 | ab | 0.415±0.039 | a | 0.168±0.013 | cd | 0.343±0.014 | b |
| **PCA** | 0.066±0.003 | a | 0.036±0.001 | b | 0.022±0.001 | c | 0.020±0.002 | c | 0.061±0.005 | a | 0.029±0.003 | b | 0.006±0.001 | d | 0.036±0.001 | b |
| **GABA** | 0.003±0.000 | f | 0.019±0.001 | b | 0.013±0.001 | cd | 0.022±0.001 | a | 0.011±0.001 | de | 0.021±0.000 | ab | 0.014±0.001 | c | 0.009±0.000 | e |
| **4Pb** | nd |  | 0.023±0.003 | a | 0.010±0.001 | b | 0.002±0.000 | c | nd |  | 0.005±0.001 | c | 0.020±0.002 | a | 0.003±0.000 | c |
| **tHB** | 0.003±0.000 | f | 0.008±0.000 | e | 0.011±0.001 | d | 0.007±0.000 | e | 0.013±0.000 | c | 0.018±0.001 | a | 0.015±0.001 | b | 0.012±0.001 | cd |
| **HAH** | 0.006±0.000 | c | 0.014±0.001 | a | 0.006±0.000 | cd | 0.005±0.001 | d | 0.008±0.001 | b | 0.006±0.000 | cd | 0.002±0.000 | e | 0.005±0.000 | cd |
| **SFu** | nd |  | nd |  | nd |  | 0.005±0.000 |  | nd |  | nd |  | nd |  | nd |  |
| **TA** | 0.169±0.009 | cd | 0.194±0.009 | c | 0.138±0.004 | d | 0.175±0.006 | cd | 0.334±0.026 | a | 0.284±0.021 | b | 0.081±0.013 | e | 0.264±0.019 | b |
| **CI** | 0.096±0.003 | c | 0.080±0.009 | cd | 0.056±0.009 | de | 0.096±0.006 | c | 0.341±0.021 | a | 0.246±0.008 | b | 0.045±0.001 | e | 0.232±0.020 | b |
| **QA** | 0.071±0.002 | d | 0.176±0.009 | ab | 0.194±0.009 | a | 0.132±0.005 | c | 0.167±0.011 | b | 0.164±0.004 | b | 0.131±0.004 | c | 0.174±0.007 | b |
| **Fru** | 1.638±0.054 | d | 5.012±0.154 | a | 5.092±0.366 | a | 5.048±0.176 | a | 2.078±0.138 | cd | 2.184±0.041 | c | 2.412±0.103 | bc | 2.720±0.140 | b |
| **Gal** | 0.064±0.003 | d | 0.144±0.016 | c | 0.178±0.007 | bc | 1.131±0.035 | a | 0.219±0.001 | b | 0.056±0.002 | d | 0.209±0.006 | b | 0.189±0.012 | b |
| **Man** | 1.276±0.019 | e | 3.533±0.071 | a | 3.096±0.222 | b | 3.096±0.052 | b | 1.661±0.018 | d | 1.536±0.038 | de | 1.723±0.057 | d | 2.003±0.087 | c |
| **Glc** | 1.542±0.026 | e | 4.231±0.129 | a | 3.781±0.098 | b | 3.469±0.248 | b | 1.751±0.022 | de | 1.782±0.078 | de | 1.921±0.055 | d | 2.285±0.089 | c |
| **RA** | 0.014±0.001 | b | 0.020±0.001 | a | 0.020±0.001 | a | 0.014±0.001 | b | 0.019±0.001 | a | 0.019±0.000 | a | 0.011±0.001 | c | 0.015±0.000 | b |
| **GA** | 0.001±0.000 | e | 0.035±0.001 | bc | 0.039±0.002 | bc | 0.048±0.001 | ab | 0.021±0.001 | d | 0.061±0.001 | a | 0.028±0.013 | cd | 0.041±0.003 | bc |
| **Dan** | 0.007±0.001 | c | 0.031±0.004 | a | 0.005±0.001 | c | 0.029±0.003 | a | 0.021±0.002 | b | 0.027±0.003 | ab | 0.001±0.000 | c | 0.030±0.003 | a |
| **Ins** | 0.813±0.011 | bc | 0.620±0.023 | c | 0.615±0.057 | c | 0.524±0.019 | c | 1.448±0.026 | a | 1.061±0.029 | ab | 0.549±0.403 | c | 0.905±0.045 | bc |
| **CA** | 0.007±0.001 | b | 0.016±0.002 | a | 0.003±0.001 | c | 0.016±0.002 | a | 0.012±0.001 | a | 0.014±0.003 | a | 0.001±0.000 | c | 0.015±0.002 | a |
| **Arb** | 0.228±0.011 | bc | 0.177±0.004 | cde | 0.133±0.006 | e | 0.191±0.015 | bcd | 0.438±0.044 | a | 0.160±0.012 | de | 0.045±0.025 | f | 0.248±0.014 | b |

*nd = not detected

**Table S2.** Quantification of detected compounds (peak area/internal standard area) in the polar extracts of stems. Different letters show significant difference between samples (*p*<0.05).

|  | **MEXV50** |  | **G3V50** |  | **G8V50** |  | **G17V50** |  | **MEXV100** |  | **G3V100** |  | **G8V100** |  | **G17V100** |  |
| --- | --- | --- | --- | --- | --- | --- | --- | --- | --- | --- | --- | --- | --- | --- | --- | --- |
| **LA** | 0.001±0.000 | abc | 0.001±0.000 | abc | 0.002±0.000 | a | 0.001±0.000 | c | 0.002±0.001 | ab | 0.001±0.000 | bc | 0.001±0.000 | abc | 0.001±0.000 | c |
| **Ala** | 0.001±0.000 | a | 0.001±0.000 | bc | 0.001±0.000 | cd | 0.001±0.000 | cd | 0.001±0.000 | b | 0.001±0.000 | de | 0.001±0.000 | de | 0.001±0.000 | e |
| **Val** | 0.001±0.000 | a | 0.001±0.000 | cd | 0.001±0.000 | cd | 0.001±0.000 | bc | 0.001±0.000 | b | 0.001±0.000 | cd | 0.001±0.000 | d | nd |  |
| **GLY** | 0.016±0.001 | a | 0.008±0.001 | c | 0.012±0.001 | b | 0.005±0.000 | d | 0.006±0.006 | cd | 0.006±0.001 | d | 0.005±0.000 | d | 0.004±0.001 | d |
| **Pro** | 0.027±0.001 | a | 0.022±0.002 | b | 0.013±0.001 | c | 0.008±0.000 | d | 0.004±0.000 | e | 0.002±0.001 | e | 0.002±0.000 | e | 0.001±0.000 | e |
| **SU** | 0.003±0.001 | ab | 0.004±0.000 | a | 0.002±0.000 | ab | 0.003±0.000 | ab | 0.003±0.000 | ab | 0.002±0.001 | b | 0.002±0.000 | ab | 0.003±0.001 | ab |
| **Ser** | nd |  | 0.001±0.000 | b | nd |  | nd |  | 0.001±0.000 | a | nd |  | nd |  | nd |  |
| **Thr** | 0.001±0.000 | a | 0.001±0.000 | b | 0.001±0.000 | c | 0.007±0.000 | b | 0.001±0.000 | b | 0.001±0.000 | c | 0.001±0.000 | cd | 0.001±0.000 | d |
| **MA** | 0.010±0.001 | b | 0.023±0.003 | a | 0.007±0.001 | b | 0.007±0.000 | b | 0.010±0.000 | b | 0.012±0.003 | b | 0.026±0.001 | a | 0.011±0.004 | b |
| **PCA** | 0.15±0.00 | a | 0.001±0.000 | b | 0.001±0.000 | bc | 0.001±0.000 | bc | 0.001±0.000 | b | 0.001±0.000 | cd | 0.001±0.000 | c | 0.001±0.000 | d |
| **GABA** | 0.001±0.000 | ab | 0.001±0.000 | b | 0.001±0.000 | bc | 0.002±0.000 | ab | 0.002±0.000 | a | 0.001±0.000 | bc | 0.001±0.000 | cd | 0.001±0.000 | d |
| **tHB** | 0.001±0.000 | d | 0.001±0.000 | cd | 0.001±0.000 | d | 0.001±0.000 | d | 0.001±0.000 | a | 0.001±0.000 | abc | 0.001 ±0.000 | ab | 0.001±0.000 | bcd |
| **HAH** | 0.001±0.001 | a | 0.001±0.000 | b | 0.001±0.000 | b | 0.001±0.000 | c | 0.001±0.000 | c | 0.001±0.000 | c | 0.001±0.000 | c | 0.001±0.000 | c |
| **TA** | 0.010±0.001 | b | 0.020±0.003 | a | 0.009±0.001 | b | 0.005±0.000 | b | 0.006±0.000 | b | 0.009±0.002 | b | 0.009±0.001 | b | 0.010±0.004 | b |
| **CI** | 0.019±0.007 | b | 0.047±0.001 | a | 0.015±0.001 | b | 0.007±0.001 | b | 0.007±0.005 | b | 0.037±0.010 | a | 0.040±0.002 | a | 0.015±0.003 | b |
| **QA** | 0.002±0.000 | d | 0.004±0.000 | a | 0.003±0.000 | c | 0.002±0.000 | c | 0.003±0.000 | bc | 0.003±0.001 | cd | 0.004±0.000 | ab | 0.003±0.000 | c |
| **Fru** | 0.176±0.016 | abc | 0.217±0.009 | a | 0.076±0.009 | c | 0.074±0.004 | c | 0.102±0.076 | bc | 0.187±0.057 | ab | 0.176±0.019 | abc | 0.175±0.028 | abc |
| **Man** | 0.062±0.008 | b | 0.089±0.005 | a | 0.050±0.001 | bcd | 0.060±0.006 | bc | 0.049±0.003 | bcd | 0.034±0.009 | d | 0.045±0.003 | cd | 0.038±0.006 | d |
| **Glc** | 0.369±0.038 | d | 0.931±0.084 | ab | 0.343±0.257 | d | 0.872±0.066 | abc | 0.612±0.042 | bcd | 0.546±0.149 | cd | 0.917±0.054 | ab | 1.080±0.173 | a |
| **Gal** | 0.003±0.000 | b | 0.004±0.000 | b | 0.002±0.000 | b | 0.005±0.000 | b | 0.003±0.000 | b | 0.003±0.001 | b | 0.074±0.010 | a | 0.006±0.001 | b |
| **Ins** | 0.036±0.002 | a | 0.020±0.002 | b | 0.012±0.001 | c | 0.006±0.000 | d | 0.019±0.002 | b | 0.005±0.001 | d | 0.007±0.000 | d | 0.006±0.001 | d |
| **Sucr** | 0.002±0.000 | c | 0.006±0.009 | bc | 0.001±0.000 | c | 0.001±0.000 | c | 0.141±0.018 | a | 0.026±0.006 | b | 0.004±0.000 | c | 0.010±0.002 | bc |
| **Mal** | 0.005±0.001 | b | 0.008±0.001 | b | 0.004±0.000 | b | 0.015±0.002 | a | 0.005±0.004 | b | 0.006±0.001 | b | 0.005±0.000 | b | 0.016±0.004 | a |

*nd = not detected

**Table S3.** Quantification of detected compounds (peak area/internal standard area) in the non-polar extracts of flowers. Different letters show significant difference between samples (*p*<0.05).

|  | **MEXV50** | | | **G3V50** | | | **G8V50** | | | **G17V50** | | | **MEXV100** | | | **G3V100** | | | **G8V100** | | | **G17V100** | | | |
| --- | --- | --- | --- | --- | --- | --- | --- | --- | --- | --- | --- | --- | --- | --- | --- | --- | --- | --- | --- | --- | --- | --- | --- | --- | --- |
| **tm Ude** | | 0.003±0.000 | c | | 0.004±0.000 | c | | 0.004±0.001 | c | | 0.028±0.000 | a | | 0.005±0.001 | c | | 0.009±0.001 | bc | | nd |  | | 0.012±0.002 | b |  |
| **OxA (16:0)** | | nd |  | | nd |  | | nd |  | | nd |  | | nd |  | | 0.041±0.010 | a | | nd |  | | nd |  |  |
| **Phy** | | nd |  | | 0.044±0.007 | d | | nd |  | | 0.039±0.004 | a | | 0.011±0.002 | c | | 0.065±0.007 | cd | | 0.065±0.004 | cd | | 0.026±0.002 | b |  |
| **C14:0** | | nd |  | | 0.052±0.004 | c | | 0.022±0.002 | d | | 0.074±0.007 | bc | | 0.081±0.015 | b | | 0.089±0.009 | b | | 0.055±0.003 | c | | 0.012±0.001 | a |  |
| **12-Me C14:0** | | 0.013±0.003 | d | | 0.064±0.007 | c | | 0.024±0.010 | d | | 0.075±0.086 | bc | | 0.089±0.024 | bc | | 0.009±0.001 | bc | | 0.007±0.001 | bc | | 0.010±0.001 | a |  |
| **2,4-DTBP** | | 0.052±0.014 | d | | 0.038±0.084 | bc | | 0.010±0.026 | d | | 0.047±0.010 | ab | | 0.030±0.005 | c | | 0.041±0.004 | bc | | 0.029±0.003 | b | | 0.061±0.005 | a |  |
| **C16:0** | | 0.214±0.097 | c | | 0.766±0.132 | b | | 0.289±0.078 | c | | 0.985±0.109 | b | | 1.048±0.280 | ab | | 0.977±0.199 | b | | 0.731±0.047 | b | | 1.408±0.094 | a |  |
| **C16:1n-7** | | nd |  | | nd |  | | 0.004±0.001 | a | | nd |  | | nd |  | | nd |  | | nd |  | | nd |  |  |
| **C16:1n-9** | | nd |  | | nd |  | | nd |  | | 0.032±0.005 | b | | 0.039±0.010 | b | | 0.026±0.005 | bc | | 0.017±0.006 | c | | 0.057±0.005 | a |  |
| **14-Me C16:0** | | 0.006±0.002 | e | | 0.033±0.002 | cd | | 0.013±0.004 | de | | 0.061±0.006 | b | | 0.061±0.016 | b | | 0.061±0.008 | b | | 0.048±0.003 | bc | | 0.089±0.007 | a |  |
| **C18:0** | | 0.116±0.024 | c | | 0.279±0.064 | ab | | 0.116±0.033 | c | | 0.259±0.038 | ab | | 0.181±0.043 | bc | | 0.264±0.025 | ab | | 0.216±0.017 | bc | | 0.333±0.030 | a |  |
| **C18:1** | | 0.032±0.006 | d | | 0.931±0.144 | a | | 0.025±0.009 | d | | 0.930±0.070 | d | | 0.162±0.040 | d | | 0.396±0.063 | c | | 0.194±0.022 | d | | 0.689±0.057 | b |  |
| **C18:2** | | 0.031±0.004 | d | | 0.374±0.038 | d | | 0.040±0.015 | d | | 2.260±0.292 | b | | 0.952±0.237 | c | | 0.955±0.122 | c | | 0.397±0.035 | d | | 2.738±0.210 | a |  |
| **C18:3** | | 0.013±0.002 | d | | 0.560±0.042 | d | | 0.026±0.011 | d | | 3.083±0.349 | b | | 1.288±0.311 | c | | 0.541±0.069 | d | | 0.507±0.044 | d | | 4.345±0.321 | a |  |
| **C20:0** | | 0.010±0.002 | d | | 0.026±0.002 | cd | | 0.008±0.003 | d | | 0.096±0.011 | b | | 0.040±0.009 | c | | 0.080±0.008 | b | | 0.032±0.001 | c | | 0.136±0.012 | a |  |
| **PE 16:0/16:0** | | 0.015±0.005 | cd | | 0.011±0.004 | cd | | 0.008±0.003 | d | | 0.027±0.003 | b | | 0.027±0.005 | b | | 0.022±0.004 | bc | | 0.042±0.003 | a | | 0.047±0.006 | a |  |

*nd = not detected

**Table S4.** Quantification of detected compounds (peak area/internal standard area) in the non-polar extracts of stems. Different letters show significant difference between samples (*p*<0.05).

|  | **MEXV50** | | **G3V50** | | | **G8V50** | | | **G17V50** | | | **MEXV100** | | | **G3V100** | | | **G8V100** | | | **G17V100** | | |
| --- | --- | --- | --- | --- | --- | --- | --- | --- | --- | --- | --- | --- | --- | --- | --- | --- | --- | --- | --- | --- | --- | --- | --- |
| **tm Ude** | nd |  | | nd |  | | nd |  | | 0.001±0.000 | a | | nd |  | | nd |  | | nd |  | | nd |  |
| **OxA (15:0)** | 0.001±0.000 | bc | | nd |  | | 0.002±0.000 | a | | nd |  | | nd |  | | 0.001±0.000 | abc | | 0.002±0.000 | ab | | 0.001±0.000 | cd |
| **C14:0** | 0.012±0.003 | bcd | | 0.016±0.001 | ab | | 0.022±0.006 | a | | 0.005±0.001 | cd | | 0.003±0.001 | d | | 0.010±0.002 | bcd | | 0.014±0.006 | abc | | 0.005±0.001 | cd |
| **C15:0** | 0.010±0.004 | bc | | 0.016±0.001 | ab | | 0.019±0.005 | a | | nd |  | | nd |  | | 0.010±0.001 | bc | | 0.016±0.005 | ab | | 0.006±0.001 | cd |
| **2,4-DTBP** | 0.003±0.001 | b | | 0.032±0.012 | bc | | 0.064±0.011 | a | | 0.007±0.002 | cd | | nd |  | | 0.033±0.005 | b | | 0.049±0.011 | ab | | 0.006±0.001 | d |
| **NA** | nd |  | | nd |  | | 0.005±0.001 | b | | 0.013±0.001 | a | | 0.005±0.003 | b | | 0.002±0.000 | bc | | 0.012±0.001 | a | | 0.003±0.000 | bc |
| **C16:0** | 0.501±0.119 | d | | 1.421±0.387 | b | | 1.177±0.179 | bc | | 0.670±0.063 | cd | | 0.557±0.149 | d | | 2.019±0.258 | a | | 1.156±0.179 | bc | | 0.422±0.062 | d |
| **C16:1n-7** | nd |  | | nd |  | | 0.024±0.002 | a | | 0.005±0.001 | cd | | nd |  | | 0.013±0.002 | bc | | nd |  | | nd |  |
| **C16:1n-9** | nd |  | | 0.024±0.004 | ab | | 0.029±0.006 | ab | | nd |  | | nd |  | | 0.010±0.001 | a | | 0.036±0.006 | a | | nd |  |
| **C18:0** | 0.169±0.028 | a | | 0.348±0.120 | a | | 0.308±0.045 | a | | 0.241±0.026 | a | | 0.264±0.119 | a | | 0.346±0.035 | a | | 0.226±0.045 | a | | 0.343±0.028 | a |
| **C18:1** | nd |  | | 0.159±0.044 | a | | 0.125±0.012 | a | | 0.042±0.011 | bc | | nd |  | | 0.044±0.007 | bc | | 0.056±0.012 | b | | 0.014±0.005 | bc |
| **C18:2** | nd |  | | 0.766±0.294 | ab | | 0.782±0.082 | ab | | 0.050±0.002 | cd | | nd |  | | 1.101±0.087 | a | | 0.432±0.082 | b | | nd |  |
| **C18:3** | nd |  | | 1.144±0.167 | b | | 1.537±0.215 | ab | | 0.040±0.007 | c | | nd |  | | 1.786±0.107 | a | | 1.179±0.215 | b | | nd |  |
| **HCtb** | nd |  | | 0.015±0.003 | b | | 0.027±0.004 | a | | 0.006±0.001 | cd | | nd |  | | 0.009±0.003 | bc | | 0.015±0.004 | a | | nd |  |
| **C20:0** | 0.009±0.001 | b | | 0.037±0.013 | bc | | 0.040±0.001 | a | | 0.024±0.003 | ab | | 0.027±0.019 | ab | | 0.034±0.003 | c | | 0.041±0.001 | abc | | 0.008±0.001 | b |

*nd = not detected
